# Supplementary material for: Modeling glioblastoma heterogeneity as a dynamic network of cell states
Source: Mol Syst Biol. 2021 Sep 16;17(9):e10105. doi: 10.15252/msb.202010105 (PMC8444284; doi:10.15252/msb.202010105)
Supplement: Supplementary file 6 — Source Data for Figure 5 [file MSB-17-e10105-s004.zip › Figure5A_sourcedata/GSEA_3017/nosigdb_stateE.GseaPreranked.1622795031093/DOROSZKO_U3013MG_CLUSTER_2.html]

Details for gene set DOROSZKO\_U3013MG\_CLUSTER\_2[GSEA]

|  || Dataset | state63017 |
| Phenotype | NoPhenotypeAvailable |
| Upregulated in class | na\_neg |
| GeneSet | DOROSZKO\_U3013MG\_CLUSTER\_2 |
| Enrichment Score (ES) | -0.73826617 |
| Normalized Enrichment Score (NES) | -4.2847795 |
| Nominal p-value | 0.0 |
| FDR q-value | 0.0 |
| FWER p-Value | 0.0 |
Table: GSEA Results Summary

  

Fig 1: Enrichment plot: DOROSZKO\_U3013MG\_CLUSTER\_2      
 Profile of the Running ES Score & Positions of GeneSet Members on the Rank Ordered List

  

| PROBE | GENE SYMBOL | GENE\_TITLE | RANK IN GENE LIST | RANK METRIC SCORE | RUNNING ES | CORE ENRICHMENT || 1 | TUBA1C |  |  | 282 | -0.253 | -0.3656 | No |
| 2 | POLR2A |  |  | 361 | -0.272 | -0.4650 | No |
| 3 | POP7 |  |  | 370 | -0.273 | -0.4732 | No |
| 4 | APOLD1 |  |  | 374 | -0.275 | -0.4748 | No |
| 5 | OIP5 |  |  | 426 | -0.291 | -0.5389 | No |
| 6 | JPT1 |  |  | 439 | -0.296 | -0.5521 | No |
| 7 | LSM8 |  |  | 441 | -0.297 | -0.5509 | No |
| 8 | ILF2 |  |  | 497 | -0.313 | -0.6200 | No |
| 9 | GGH |  |  | 504 | -0.314 | -0.6252 | No |
| 10 | HNRNPH3 |  |  | 507 | -0.316 | -0.6252 | No |
| 11 | PGP |  |  | 512 | -0.318 | -0.6278 | No |
| 12 | KIF20A |  |  | 523 | -0.321 | -0.6382 | No |
| 13 | RHEB |  |  | 528 | -0.323 | -0.6407 | No |
| 14 | ACTL6A |  |  | 533 | -0.325 | -0.6432 | No |
| 15 | GPSM2 |  |  | 550 | -0.334 | -0.6613 | No |
| 16 | SAP30 |  |  | 558 | -0.339 | -0.6676 | No |
| 17 | FAM83D |  |  | 559 | -0.340 | -0.6648 | No |
| 18 | TROAP |  |  | 562 | -0.341 | -0.6646 | No |
| 19 | LSM5 |  |  | 568 | -0.342 | -0.6683 | No |
| 20 | CKLF |  |  | 574 | -0.347 | -0.6719 | No |
| 21 | HMGB3 |  |  | 577 | -0.348 | -0.6716 | No |
| 22 | CACYBP |  |  | 589 | -0.352 | -0.6830 | No |
| 23 | DDX39A |  |  | 590 | -0.353 | -0.6801 | No |
| 24 | CDK5RAP2 |  |  | 591 | -0.353 | -0.6772 | No |
| 25 | HIRIP3 |  |  | 603 | -0.359 | -0.6885 | No |
| 26 | ZWILCH |  |  | 604 | -0.360 | -0.6856 | No |
| 27 | POC1A |  |  | 611 | -0.363 | -0.6904 | No |
| 28 | BTG3 |  |  | 615 | -0.365 | -0.6913 | No |
| 29 | G2E3 |  |  | 624 | -0.371 | -0.6986 | No |
| 30 | NEK2 |  |  | 628 | -0.374 | -0.6994 | No |
| 31 | EXOSC8 |  |  | 630 | -0.376 | -0.6976 | No |
| 32 | CCP110 |  |  | 636 | -0.381 | -0.7010 | No |
| 33 | CDKN2D |  |  | 638 | -0.381 | -0.6991 | No |
| 34 | PHIP |  |  | 643 | -0.384 | -0.7011 | No |
| 35 | PHF19 |  |  | 648 | -0.391 | -0.7031 | No |
| 36 | TEDC1 |  |  | 651 | -0.394 | -0.7024 | No |
| 37 | SHCBP1 |  |  | 655 | -0.398 | -0.7030 | No |
| 38 | MNS1 |  |  | 666 | -0.408 | -0.7127 | No |
| 39 | SOGA1 |  |  | 672 | -0.411 | -0.7158 | No |
| 40 | NUDT1 |  |  | 674 | -0.413 | -0.7137 | No |
| 41 | SAC3D1 |  |  | 679 | -0.416 | -0.7154 | No |
| 42 | CENPQ |  |  | 689 | -0.425 | -0.7236 | No |
| 43 | NSD2 |  |  | 693 | -0.426 | -0.7240 | No |
| 44 | CKAP5 |  |  | 694 | -0.426 | -0.7205 | No |
| 45 | DIAPH3 |  |  | 696 | -0.427 | -0.7183 | No |
| 46 | ANP32B |  |  | 699 | -0.431 | -0.7173 | No |
| 47 | PARPBP |  |  | 705 | -0.434 | -0.7202 | No |
| 48 | LMNB2 |  |  | 713 | -0.438 | -0.7257 | No |
| 49 | ALYREF |  |  | 716 | -0.444 | -0.7246 | No |
| 50 | MASTL |  |  | 721 | -0.451 | -0.7261 | No |
| 51 | TTK |  |  | 724 | -0.454 | -0.7249 | No |
| 52 | RAD21 |  |  | 728 | -0.458 | -0.7250 | No |
| 53 | SPAG5 |  |  | 729 | -0.458 | -0.7212 | No |
| 54 | LBR |  |  | 739 | -0.463 | -0.7291 | No |
| 55 | TK1 |  |  | 747 | -0.480 | -0.7343 | Yes |
| 56 | KNSTRN |  |  | 751 | -0.485 | -0.7342 | Yes |
| 57 | PRR11 |  |  | 755 | -0.488 | -0.7340 | Yes |
| 58 | SPDL1 |  |  | 758 | -0.491 | -0.7326 | Yes |
| 59 | CDCA3 |  |  | 759 | -0.497 | -0.7284 | Yes |
| 60 | TRIP13 |  |  | 760 | -0.498 | -0.7243 | Yes |
| 61 | MXD3 |  |  | 763 | -0.502 | -0.7227 | Yes |
| 62 | GMNN |  |  | 766 | -0.510 | -0.7211 | Yes |
| 63 | RTKN2 |  |  | 767 | -0.511 | -0.7169 | Yes |
| 64 | DTYMK |  |  | 774 | -0.522 | -0.7204 | Yes |
| 65 | CENPA |  |  | 782 | -0.538 | -0.7250 | Yes |
| 66 | SKA3 |  |  | 783 | -0.539 | -0.7205 | Yes |
| 67 | NCAPD2 |  |  | 784 | -0.540 | -0.7161 | Yes |
| 68 | RPA3 |  |  | 788 | -0.550 | -0.7154 | Yes |
| 69 | NCAPD3 |  |  | 790 | -0.559 | -0.7121 | Yes |
| 70 | ATAD5 |  |  | 791 | -0.559 | -0.7074 | Yes |
| 71 | CDC20 |  |  | 793 | -0.562 | -0.7041 | Yes |
| 72 | RACGAP1 |  |  | 796 | -0.566 | -0.7020 | Yes |
| 73 | ASF1B |  |  | 798 | -0.568 | -0.6986 | Yes |
| 74 | RFC3 |  |  | 801 | -0.569 | -0.6965 | Yes |
| 75 | TUBB4B |  |  | 804 | -0.573 | -0.6943 | Yes |
| 76 | CENPN |  |  | 805 | -0.574 | -0.6895 | Yes |
| 77 | USP1 |  |  | 806 | -0.575 | -0.6848 | Yes |
| 78 | CDCA8 |  |  | 808 | -0.585 | -0.6812 | Yes |
| 79 | PLK1 |  |  | 809 | -0.586 | -0.6764 | Yes |
| 80 | PSMC3IP |  |  | 811 | -0.588 | -0.6728 | Yes |
| 81 | FOXM1 |  |  | 814 | -0.593 | -0.6705 | Yes |
| 82 | DEPDC1 |  |  | 815 | -0.596 | -0.6655 | Yes |
| 83 | CDCA2 |  |  | 816 | -0.597 | -0.6606 | Yes |
| 84 | LMNB1 |  |  | 818 | -0.606 | -0.6568 | Yes |
| 85 | MND1 |  |  | 821 | -0.615 | -0.6543 | Yes |
| 86 | AURKA |  |  | 824 | -0.623 | -0.6518 | Yes |
| 87 | CKAP2L |  |  | 826 | -0.625 | -0.6479 | Yes |
| 88 | CCDC34 |  |  | 827 | -0.626 | -0.6427 | Yes |
| 89 | DNAJC9 |  |  | 828 | -0.633 | -0.6374 | Yes |
| 90 | AURKB |  |  | 829 | -0.634 | -0.6322 | Yes |
| 91 | PIMREG |  |  | 833 | -0.641 | -0.6308 | Yes |
| 92 | BUB3 |  |  | 834 | -0.641 | -0.6254 | Yes |
| 93 | CENPW |  |  | 835 | -0.643 | -0.6201 | Yes |
| 94 | CDKN3 |  |  | 836 | -0.653 | -0.6147 | Yes |
| 95 | BUB1 |  |  | 837 | -0.653 | -0.6093 | Yes |
| 96 | ITGB3BP |  |  | 838 | -0.661 | -0.6038 | Yes |
| 97 | ANP32E |  |  | 841 | -0.676 | -0.6008 | Yes |
| 98 | KIF22 |  |  | 842 | -0.678 | -0.5951 | Yes |
| 99 | BIRC5 |  |  | 843 | -0.679 | -0.5895 | Yes |
| 100 | CENPM |  |  | 844 | -0.682 | -0.5839 | Yes |
| 101 | NCAPH |  |  | 845 | -0.685 | -0.5782 | Yes |
| 102 | BRCA1 |  |  | 846 | -0.691 | -0.5724 | Yes |
| 103 | ZWINT |  |  | 847 | -0.692 | -0.5667 | Yes |
| 104 | SPC24 |  |  | 848 | -0.694 | -0.5609 | Yes |
| 105 | CENPH |  |  | 849 | -0.694 | -0.5552 | Yes |
| 106 | DHFR |  |  | 851 | -0.701 | -0.5507 | Yes |
| 107 | CIP2A |  |  | 853 | -0.712 | -0.5460 | Yes |
| 108 | ECT2 |  |  | 854 | -0.717 | -0.5401 | Yes |
| 109 | KIF2C |  |  | 855 | -0.721 | -0.5341 | Yes |
| 110 | NUF2 |  |  | 856 | -0.721 | -0.5281 | Yes |
| 111 | CENPK |  |  | 857 | -0.722 | -0.5221 | Yes |
| 112 | FAM111A |  |  | 858 | -0.727 | -0.5161 | Yes |
| 113 | ANLN |  |  | 860 | -0.733 | -0.5113 | Yes |
| 114 | KIF4A |  |  | 861 | -0.736 | -0.5052 | Yes |
| 115 | FEN1 |  |  | 862 | -0.736 | -0.4991 | Yes |
| 116 | HMMR |  |  | 863 | -0.740 | -0.4929 | Yes |
| 117 | CCNB2 |  |  | 865 | -0.745 | -0.4881 | Yes |
| 118 | RRM1 |  |  | 866 | -0.749 | -0.4819 | Yes |
| 119 | ARHGAP11A |  |  | 867 | -0.756 | -0.4756 | Yes |
| 120 | NDC80 |  |  | 869 | -0.762 | -0.4706 | Yes |
| 121 | ORC6 |  |  | 870 | -0.766 | -0.4642 | Yes |
| 122 | BUB1B |  |  | 871 | -0.766 | -0.4578 | Yes |
| 123 | CCNA2 |  |  | 872 | -0.770 | -0.4514 | Yes |
| 124 | KIF23 |  |  | 873 | -0.772 | -0.4450 | Yes |
| 125 | MELK |  |  | 874 | -0.776 | -0.4386 | Yes |
| 126 | TMPO |  |  | 875 | -0.778 | -0.4321 | Yes |
| 127 | KIF20B |  |  | 876 | -0.779 | -0.4257 | Yes |
| 128 | CEP55 |  |  | 877 | -0.780 | -0.4192 | Yes |
| 129 | CKAP2 |  |  | 878 | -0.781 | -0.4127 | Yes |
| 130 | PTTG1 |  |  | 880 | -0.791 | -0.4075 | Yes |
| 131 | H2AFX |  |  | 882 | -0.799 | -0.4021 | Yes |
| 132 | KIFC1 |  |  | 883 | -0.807 | -0.3954 | Yes |
| 133 | KIF14 |  |  | 885 | -0.809 | -0.3900 | Yes |
| 134 | EZH2 |  |  | 886 | -0.810 | -0.3833 | Yes |
| 135 | UBE2S |  |  | 887 | -0.815 | -0.3765 | Yes |
| 136 | SGO1 |  |  | 889 | -0.822 | -0.3710 | Yes |
| 137 | KIF15 |  |  | 890 | -0.834 | -0.3641 | Yes |
| 138 | CDCA5 |  |  | 892 | -0.841 | -0.3584 | Yes |
| 139 | RAD51AP1 |  |  | 894 | -0.853 | -0.3526 | Yes |
| 140 | KPNA2 |  |  | 895 | -0.858 | -0.3455 | Yes |
| 141 | DBF4 |  |  | 896 | -0.864 | -0.3383 | Yes |
| 142 | SMC2 |  |  | 897 | -0.867 | -0.3311 | Yes |
| 143 | SGO2 |  |  | 898 | -0.869 | -0.3239 | Yes |
| 144 | CENPE |  |  | 899 | -0.873 | -0.3167 | Yes |
| 145 | PBK |  |  | 900 | -0.880 | -0.3094 | Yes |
| 146 | FANCI |  |  | 901 | -0.882 | -0.3020 | Yes |
| 147 | SMC4 |  |  | 902 | -0.886 | -0.2947 | Yes |
| 148 | MIS18BP1 |  |  | 904 | -0.889 | -0.2886 | Yes |
| 149 | C21orf58 |  |  | 905 | -0.893 | -0.2812 | Yes |
| 150 | CCNB1 |  |  | 906 | -0.895 | -0.2738 | Yes |
| 151 | CKS1B |  |  | 909 | -0.912 | -0.2688 | Yes |
| 152 | HJURP |  |  | 910 | -0.921 | -0.2612 | Yes |
| 153 | CKS2 |  |  | 911 | -0.922 | -0.2535 | Yes |
| 154 | GTSE1 |  |  | 912 | -0.941 | -0.2457 | Yes |
| 155 | KIF11 |  |  | 913 | -0.957 | -0.2377 | Yes |
| 156 | MYBL2 |  |  | 914 | -0.959 | -0.2298 | Yes |
| 157 | KNL1 |  |  | 915 | -0.963 | -0.2218 | Yes |
| 158 | CLSPN |  |  | 916 | -0.964 | -0.2138 | Yes |
| 159 | TYMS |  |  | 917 | -0.965 | -0.2058 | Yes |
| 160 | CENPU |  |  | 918 | -0.971 | -0.1977 | Yes |
| 161 | PRC1 |  |  | 919 | -0.980 | -0.1896 | Yes |
| 162 | MAD2L1 |  |  | 921 | -0.990 | -0.1827 | Yes |
| 163 | UBE2T |  |  | 922 | -0.994 | -0.1744 | Yes |
| 164 | DLGAP5 |  |  | 923 | -0.998 | -0.1661 | Yes |
| 165 | SPC25 |  |  | 924 | -1.033 | -0.1576 | Yes |
| 166 | ESCO2 |  |  | 926 | -1.067 | -0.1500 | Yes |
| 167 | UBE2C |  |  | 928 | -1.108 | -0.1421 | Yes |
| 168 | NUSAP1 |  |  | 929 | -1.112 | -0.1329 | Yes |
| 169 | TPX2 |  |  | 930 | -1.124 | -0.1236 | Yes |
| 170 | NCAPG |  |  | 931 | -1.142 | -0.1141 | Yes |
| 171 | ASPM |  |  | 932 | -1.146 | -0.1046 | Yes |
| 172 | ATAD2 |  |  | 933 | -1.162 | -0.0949 | Yes |
| 173 | CENPF |  |  | 935 | -1.200 | -0.0863 | Yes |
| 174 | HMGB2 |  |  | 937 | -1.238 | -0.0773 | Yes |
| 175 | CDK1 |  |  | 938 | -1.248 | -0.0669 | Yes |
| 176 | RRM2 |  |  | 940 | -1.403 | -0.0566 | Yes |
| 177 | MKI67 |  |  | 941 | -1.407 | -0.0449 | Yes |
| 178 | TOP2A |  |  | 942 | -1.417 | -0.0331 | Yes |
| 179 | PCLAF |  |  | 943 | -1.554 | -0.0202 | Yes |
| 180 | HIST1H4C |  |  | 946 | -2.752 | -0.0000 | Yes |
Table: GSEA details [plain text format]

  

Fig 2: DOROSZKO\_U3013MG\_CLUSTER\_2: Random ES distribution      
 Gene set null distribution of ES for **DOROSZKO\_U3013MG\_CLUSTER\_2**
